# Characterization of Vemurafenib-Resistant Melanoma Cell Lines Reveals Novel Hallmarks of Targeted Therapy Resistance

**DOI:** 10.3390/ijms23179910

**Published:** 2022-08-31

**Authors:** Martina Radić, Ignacija Vlašić, Maja Jazvinšćak Jembrek, Anđela Horvat, Ana Tadijan, Maja Sabol, Marko Dužević, Maja Herak Bosnar, Neda Slade

**Affiliations:** 1 Laboratory for Protein Dynamics, Division of Molecular Medicine, Ruđer Bošković Institute, Bijenička 54, 10000 Zagreb, Croatia; 2 School of Medicine, Catholic University of Croatia, 10000 Zagreb, Croatia; 3 Laboratory for Hereditary Cancer, Division of Molecular Medicine, Ruđer Bošković Institute, 10000 Zagreb, Croatia

**Keywords:** melanoma, vemurafenib, drug resistance, signaling pathways, epithelial–mesenchymal transition (EMT), slow-cycling cells, NME metastasis suppressor proteins

## Abstract

Regardless of the significant improvements in treatment of melanoma, the majority of patients develop resistance whose mechanisms are still not completely understood. Hence, we generated and characterized two melanoma-derived cell lines, primary WM793B and metastatic A375M, with acquired resistance to the RAF inhibitor vemurafenib. The morphology of the resistant primary WM793B melanoma cells showed EMT-like features and exhibited a hybrid phenotype with both epithelial and mesenchymal characteristics. Surprisingly, the vemurafenib-resistant melanoma cells showed a decreased migration ability but also displayed a tendency to collective migration. Signaling pathway analysis revealed the reactivation of MAPK and the activation of the PI3K/AKT pathway depending on the vemurafenib-resistant cell line. The acquired resistance to vemurafenib caused resistance to chemotherapy in primary WM793B melanoma cells. Furthermore, the cell-cycle analysis and altered levels of cell-cycle regulators revealed that resistant cells likely transiently enter into cell cycle arrest at the G0/G1 phase and gain slow-cycling cell features. A decreased level of NME1 and NME2 metastasis suppressor proteins were found in WM793B-resistant primary melanoma, which is possibly the result of vemurafenib-acquired resistance and is one of the causes of increased PI3K/AKT signaling. Further studies are needed to reveal the vemurafenib-dependent negative regulators of NME proteins, their role in PI3K/AKT signaling, and their influence on vemurafenib-resistant melanoma cell characteristics.

## 1. Introduction

Melanoma is a type of cancer mostly localized in the skin (i.e., cutaneous melanoma) and can occur rarely in the eye (i.e., uveal melanoma) and mucosal membranes (i.e., mucosal melanoma) [1]. Melanoma develops from melanocytes—pigment-producing cells. It is considered to be one of the most aggressive human tumors, primarily because of its ability to spread from a relatively small primary tumor and metastasize to multiple sites [2]. Melanocytes are derived from the neural crest, a highly migratory, multipotent cell population that forms many specialized structures and tissues in the developing embryo through migration, proliferation, and differentiation [3].

This could be one of the reasons why melanoma is extremely aggressive and prone to metastasis. Prior to metastasis, melanocytes adopt a mesenchymal phenotype through the process of phenotype switching [4]. Even though this process is fairly similar to the epithelial–mesenchymal transition (EMT) and includes EMT-related genes, sometimes the term phenotype switching is used in order to highlight the non-epithelial origin of melanocytes [4,5]. The expression of mesenchymal proteins increases the motility, invasiveness, and metastatic potential of melanoma. The RAS/RAF/MEK/ERK, PI3K/AKT/mTOR, and Wnt/β-catenin pathways play key roles in promoting mesenchymal protein expression [6].

The current therapeutic options for cutaneous melanoma patients mainly consist of surgical resection, chemotherapy, immunotherapy, and targeted therapy. Surgery is the main treatment for accessible and early-stage melanoma tumors, while chemotherapy is usually applied for advanced melanoma patients with refractory, progressive, or recurrent disease [7]. In other patients, the first-line treatment depends on the BRAF mutation status. BRAF-mutated melanoma patients can receive targeted therapies or immunotherapies, while BRAF wild-type melanoma patients receive immunotherapies. Targeted therapies are preferred for BRAF-mutated melanoma patients because the response to immunotherapy may take longer [8].

Approximately 50% of cutaneous melanoma cases carry BRAF mutations of which over 90% are BRAF V600E mutations [9]. In 2011, vemurafenib was the first BRAF inhibitor (BRAFi) approved by the FDA for patients with advanced melanoma [10]. In the following years, two additional inhibitors were approved. Although these inhibitors initially showed excellent response, the long-term success was limited due to the rapid onset of resistance to treatment.

Previous studies have shown that reactivation of the MAPK pathway occurs in the majority of BRAFi-resistant tumors, suggesting that tumor cells are highly dependent on this pathway and rapidly adapt to its inhibition. The main mechanisms of MAPK reactivation include alterations in BRAF, NRAS, MEK, and neurofibromin 1 (NF1) [11,12]. The second most frequently activated pathway is PI3K/AKT. Increased PI3K/AKT signaling is usually due to loss of function of PTEN or upregulation of receptor tyrosine kinases (RTKs) [13,14].

In this study, we aim to further investigate the mechanisms of cutaneous melanoma resistance to targeted therapies. We generated melanoma cells resistant to vemurafenib from primary and metastatic cell lines, both with BRAF V600E mutations. We characterized vemurafenib-resistant melanoma cells by examining changes in the phenotype, expression of EMT markers, migration, proliferation, cell cycle, and activation of signaling pathways involved in the development of resistance.

## 2. Results and Discussion

### 2.1. A375M and WM793B Melanoma Cell Lines Develop Resistance upon Prolonged Vemurafenib Treatment

**Figure 1:**
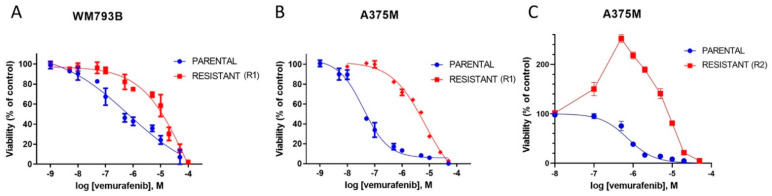
Shift in IC_50_ values following acquired resistance to vemurafenib in WM793B and A375M cell lines. WM793B (**A**) and A375M (**B**) parental and resistant cell lines were exposed to serial dilutions of vemurafenib (from 1 nM to 50 μM) for 72 h and cell viability was assessed by MTT assay. Vemurafenib-resistant melanoma cell lines WM793B-R1 and A375M-R1 showed 33-fold and 224-fold increased resistance to vemurafenib, respectively. Prolonged treatment of A375M cell line with vemurafenib (7 months, R2) resulted in peak viability of resistant cells at the concentration of vemurafenib at which they were grown (**C**). Each curve represents the mean ± SD from three to four independent experiments performed in quadruplets for each vemurafenib concentration.

The growth inhibitory effect of vemurafenib, the BRAFi, was first determined in the naive parental melanoma cell lines A375M and WM793B. In both cell lines, vemurafenib exhibited potent, concentration-dependent antiproliferative activity. Under selective pressure in the presence of vemurafenib for 8 weeks, the resulting melanoma cell lines A375M-R1 and WM793B-R1 had pronounced resistance to the antiproliferative effect of vemurafenib. In A375M cells, the IC_50_ value shifted from 0.0319 ± 0.007 μM in the naive parental cells to 7.167 ± 0.75 μM in the vemurafenib-resistant cells, representing a 224-fold increase, while WM793B cells had an IC_50_ value of 0.626 ± 0.21 μM in the naive cells and 20.50 ± 12.5 μM in the resistant cells, indicating a 33-fold change in sensitivity to the vemurafenib (Figure 1A,B).

However, prolonged treatment of the A375M cell line in vemurafenib for 7 months (R2) displayed peak viability at the vemurafenib concentration in which they were grown resulting in a bell-shaped survival curve (Figure 1C). Thus, the IC_50_ values were difficult to determine. This result suggests that the proliferation of vemurafenib-resistant cells may be dependent on the continuous presence of the drug. The bell-shaped viability curve of melanoma cells grown in vemurafenib has also been reported by others [15]. Das Thakur and co-authors observed such a dependence in melanomas expressing the oncoprotein BRAF V600E in mice.

They demonstrated that vemurafenib-resistant melanoma cells become dependent on the drug for their continued proliferation. Likewise, Rowdo and co-authors reported that the melanoma cell subpopulation that survived prolonged treatment with vemurafenib showed quiescent/senescent cancer stem-cell-like characteristics and resistance plasticity, i.e., the cells can revert to the features of parental cells upon vemurafenib withdrawal [16,17]. Some authors considered melanoma cells resistant even if the difference between the IC_50_ values of the parental and resistant cells was not found, due to other characteristics, such as increased ERK phosphorylation or a change in phenotype from epithelial to elongated and spindle-shaped [18].

### 2.2. Generated Vemurafenib-Resistant Melanoma Cell Lines Show Morphological and Molecular Changes

**Figure 2:**
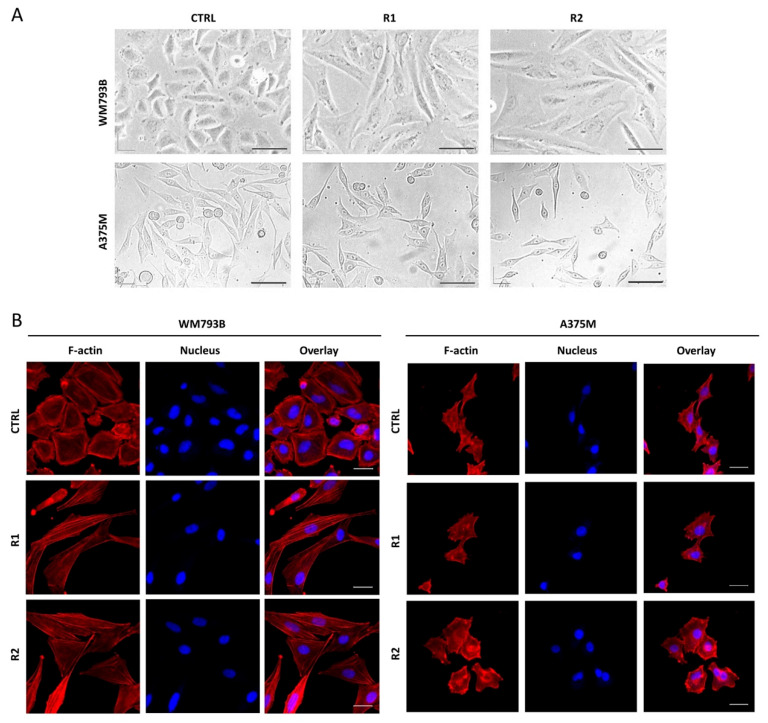
Morphology of primary melanoma cells changed upon vemurafenib treatment. Micrographs show parental (CTRL) and resistant (R) melanoma cells visualized by phase-contrast (**A**) and confocal fluorescent (**B**) microscopy. R1 and R2 represent the cells treated with vemurafenib for 4 and 9 months, respectively. F-actin is visualized with phalloidin-TRITC (red) and DNA with DAPI (blue). The scale bar is set at 150 μm (**A**) and 30 μm (**B**).

Melanoma cells are not only able to rapidly adapt to therapies by acquiring mutations; however, they also tend to alter their molecular and cellular phenotype in an EMT-like manner to evade drug treatment [8,19]. This process of cellular plasticity is driven by EMT-inducing transcription factors, mainly the SNAIL, TWIST, and ZEB protein families [20]. Indeed, we observed that vemurafenib-resistant primary melanoma cells (WM793B) changed their morphology from epithelial to spindle-shaped mesenchymal (Figure 2A).

Since the cell shape is controlled by modulation of the actin cytoskeleton [21], we visualized the actin filaments with fluorescently labeled phalloidin (Figure 2B). The parental cells showed dense cortical actin filaments, whereas the resistant cells had a high number of stress fibers, emphasizing the elongated shape of the cells. This type of phenotypic change is a common event in drug-resistant melanomas [18,22], however, has also been observed in a number of other tumors [23,24]. Several signaling pathways that regulate quiescence/dormancy, cancer stemness or EMT might be involved in such an apparent phenotype change [25,26].

EMT is a process in which epithelial cells lose their cell polarity and cell–cell adhesion and acquire migratory and invasive properties [27]. It was first recognized in embryonic development as a process during which cells lose all their epithelial characteristics and acquire a mesenchymal phenotype [28]. In cancer progression, on the other hand, cells originating from epithelial cells can exhibit both mesenchymal and epithelial characteristics. That process is known as partial EMT and is thought to enhance the invasive properties of cancer cells, generate cancer stem cells and circulating tumor cells, and promote resistance to anti-cancer drugs [29].

**Figure 3:**
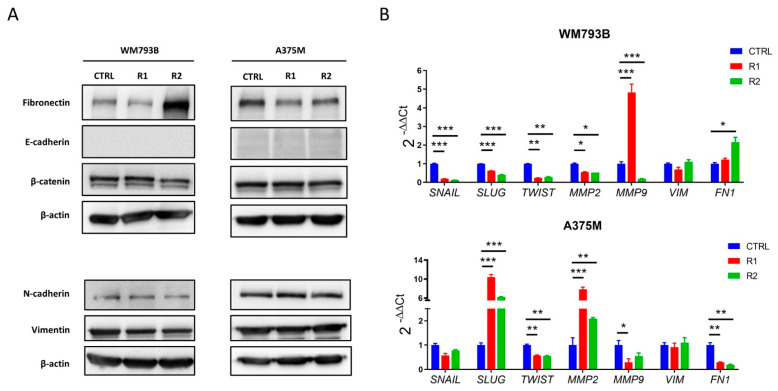
EMT marker expression in parental (CTRL) and resistant (R) WM793B and A375M cell lines. (**A**) The expression of EMT markers at the protein level was investigated by western blotting. β-actin was used as a loading control. (**B**) The gene expression levels of EMT markers were measured by q-PCR. The D’Agostino–Pearson test was used to confirm the normal distribution of continuous variables, while the parametric statistical test one-way ANOVA with the Tukey Kramer post hoc method was used to determine a statistically significant difference in the expression between subgroups. Statistical analysis was performed using MedCalc (v. 18.11.3). Statistically significant results are marked by an asterisk (*). A *p*-value less than 0.05 is flagged with one star (*), a *p*-value less than 0.01 is flagged with two stars (**), and *p*-value less than 0.001 is flagged with three stars (***).

The parental cells we used are tumor cells in which the partial EMT process may have already occurred, particularly in metastatic melanoma cells, A375M. In concordance with these findings, we assumed that our parental and resistant melanoma cells might exhibit a hybrid phenotype (both epithelial and mesenchymal characteristics). Our results showed that one of the main features of the EMT process, the loss of E-cadherin expression [30], occurred in our melanoma cell model prior to vemurafenib treatment. Both parental and resistant cells showed no expression of E-cadherin at the protein level (Figure 3A).

In addition, we did not observe an increase in the expression of the mesenchymal proteins, N-cadherin and vimentin in resistant compared to parental cells. However, we did observe a significant increase in fibronectin expression in resistant primary WM793B cells compared to parental cells. Furthermore, we investigated the gene expression of the transcription factors SNAIL, SLUG, and TWIST, which repress E-cadherin [31,32,33] (Figure 3B).

In the primary melanoma cell line, WM793B, we observed a decrease in the expression of all three genes in resistant cells compared to parental cells, while in the metastatic cell line, A375M, we observed an increase in the expression of the *SLUG* gene. In addition, we observed upregulation of matrix metalloproteinase-2 (MMP2) at the gene level in resistant metastatic melanoma cell lines (A375M R1 and R2) compared to the parental cell line (A375M CTRL). Previous studies have shown that cancer cells that undergo EMT show an increase in MMP-2 expression, which facilitates cell invasion and metastasis [34,35].

Furthermore, we observed upregulation of matrix metalloproteinase-9 (MMP-9) in the resistant primary melanoma cell line (WM793B R1) compared to the parental line (WM793B CTRL), while prolonged treatment with vemurafenib (WM793B R2) decreased the protein levels of MMP-9. It has been known that overexpression of MMP-9 promotes melanoma invasiveness and spreading via the degradation of several components of the extracellular matrix [36,37,38]. Our results do not provide a clear picture of the changes associated with the EMT process. However, this is to be expected since EMT is a complex process and sustains cell plasticity by causing changes in many signaling pathways.

### 2.3. Migration Capacity of Melanoma Cells Decreases with the Onset of Vemurafenib-Resistance

**Figure 4:**
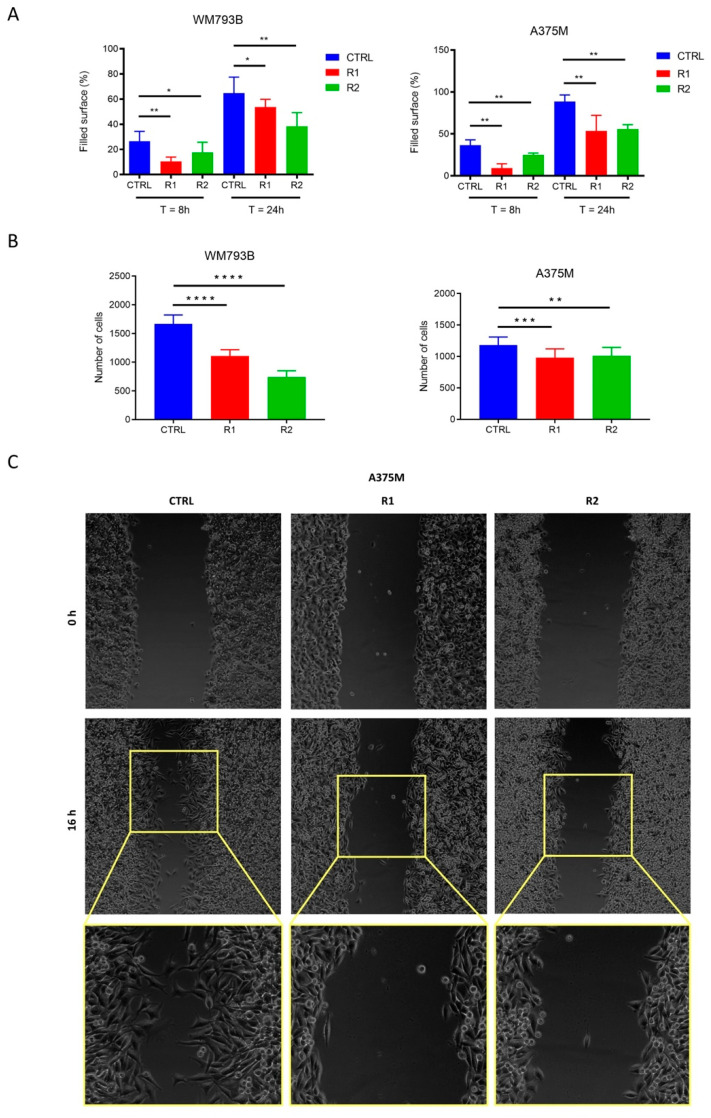
Migration of parental and resistant WM793B and A375M melanoma cells. For the wound healing assay (**A**), parental cells (CTRL) and vemurafenib-resistant sublines (R1 and R2) were imaged at the time of causing the wound and 8 and 24 h after. The results are shown as a filled area at a given time point. The mean and standard deviation of 12 measurements for each sample and time point are shown. For the culture insert assay (**B**), parental cells (CTRL) and vemurafenib-resistant sublines (R1 and R2) were imaged one hour after seeding in the upper chamber. The results are presented as the number of cells passing the membrane from the upper to the lower chamber. The mean and standard deviation of six measurements per sample are shown. The Mann–Whitney test (GraphPad Prism v. 7.04) was used for statistical analysis. Statistically significant results are marked by an asterisk (*). A *p*-value less than 0.05 is flagged with one star (*), a *p*-value less than 0.01 is flagged with two stars (**), a *p*-value less than 0.001 is flagged with three stars (***), and if a *p*-value is less than 0.0001, it is flagged with four stars (****). Micrograph of the wound healing assay of A375M melanoma cells after 16 h filmed with video microscopy (**C**). The parental A375M cells (CTRL) close the wound by single-cell migration, whereas the vemurafenib-resistant cells (R1 and R2) exhibit collective migration.

Using cell migration assays, a decreased migration ability of vemurafenib-resistant cells compared to parental cells was observed (Figure 4A,B). The obtained results are especially intriguing as previous studies have linked the drug resistance to increased migration of melanoma cells [18,39] as well as other tumor types [40,41].

Using video microscopy, we identified additional differences in the migration between A375M parental and vemurafenib-resistant cells. We observed that parental cells fill the wound by single-cell migration (Figure 4C), with each cell migrating in a different direction and at a different rate, thereby, filling the wound rapidly. Resistant cells, on the other hand, fill up the wound by collective migration. The direction of each cell depends on the neighboring cells and the front of a collective of migrating cells is clearly visible.

Time-lapse video microscopy of this process is available in the Supplementary Data (Video S1). It has been shown that metastasis as a result of single-cell migration requires EMT modifications, whereas intravasation by collective cell migration is not necessarily dependent on the EMT process [42]. In this non-EMT mediated mode of metastasis, caused by collective migration, invasive leader cells are expressing E-cadherin and basal epithelial markers, such as cytokeratin 14 and p63 [43]. Collective cell migration has been shown to have a higher invasive capacity and resistance to clinical treatments compared with single-cell migration [44].

However, as with EMT, the switch from individual to collective migration and back is quite fluid [45]. As reviewed in Campbell and Casanova [46], it is clearly not possible to place the cellular migration process strictly in one of the categories. It seems that during the migration process the cells are transiting between different stages and that only extreme cases can be defined exclusively as migrating single cells that underwent EMT or cells that migrate collectively. This is more visible in the case of cancer cells where cells rapidly adapt to new environments and circumstances to acquire robustness, speed, and plasticity.

### 2.4. MAPK and PI3K Pathways Play a Significant Role in the Occurrence of Resistance

**Figure 5:**
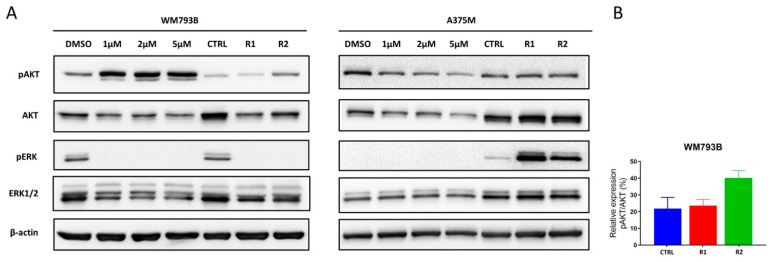
AKT and ERK phosphorylation/activation in vemurafenib treated and resistant melanoma cell lines. (**A**) The activity of signaling pathways in parental lines (CTRL) after short-term 24 h treatment with vemurafenib (1, 2, and 5 μM) or DMSO, and in cell lines resistant to vemurafenib (R1 and R2) is analyzed by western blot. β-actin was used as a loading control. (**B**) Densitometric analysis of pAKT/AKT signals from two biological replicates shows activation of PI3K/AKT signaling pathway in vemurafenib-resistant primary melanoma cell line (WM793B-R).

To further characterize vemurafenib-resistant cell lines, we examined the role of MAPK and PI3K/AKT signaling pathways in the occurrence of resistance. As previously mentioned, the activation of these signaling pathways presents a common mechanism of resistance to targeted therapy in melanoma [7]. Our results showed a significant activation of the PI3K/AKT pathway in primary WM793B melanoma cells after a short-term, 2-h treatment with vemurafenib, visible by a significant increase in pAKT levels (Figure 5A).

In addition, densitometric analysis showed that the pAKT/AKT ratio is higher in resistant cells (23.6% in R1 and 40.2% in R2) compared to parental cells (21.9% in CTRL), confirming the activation of the PI3K/AKT pathway in WM793B vemurafenib-resistant cells (Figure 5B). On the other hand, the significant increase in pERK expression in resistant A375M cells compared to parental (Figure 5A) implies that the development of resistance in metastatic melanoma (A375M cell line) is associated with a strong reactivation of the MAPK pathway. As expected, the short-term treatment with vemurafenib at concentrations of 1, 2, and 5 µM for 24 h decreased the expression of pERK to an extent that we could no longer detect the signal in both cell lines (Figure 5A), thus, confirming the potent effect of BRAFi.

### 2.5. Vemurafenib-Resistant Primary Melanoma Cell Line WM793B Enters a Slow-Cycling State

**Figure 6:**
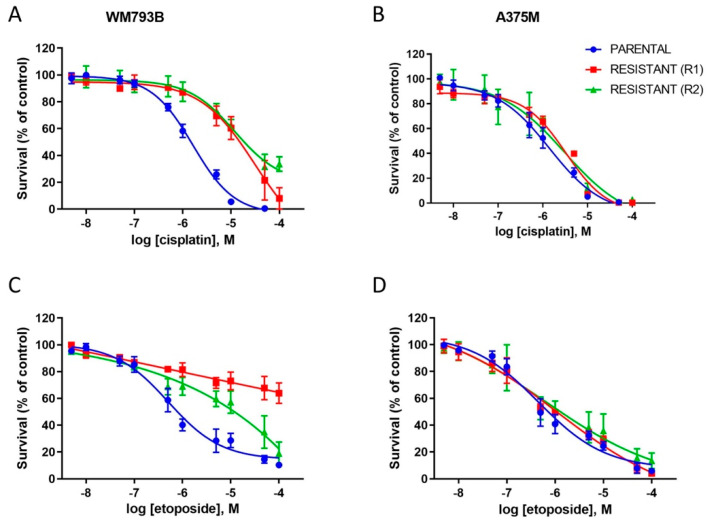
Differential sensitivity of parental and resistant WM793B and A375M cell lines to cisplatin and etoposide. WM793B and A375M parental and resistant cells were exposed to serial dilutions of cisplatin and etoposide for 72 h, and the cell viability was assessed by MTT assay. Vemurafenib-resistant WM793B cells increased tolerance to both cisplatin (**A**) and etoposide (**C**). On the other hand, vemurafenib-resistant A375M cells showed similar resistance to cisplatin (**B**) and etoposide (**D**) as parental cells. Each dose–response curve represents the mean ± SD from three to five independent experiments.

One of the mechanisms of resistance to therapy is the transition of melanoma cells into a slow-cycling state [26,47,48]. It has been shown that vemurafenib treatment also leads to the enrichment of highly invasive slow-cycling melanoma cells [49,50]. In addition, slow-cycling cells (SCCs) manifest resistance to various other treatments [26]. To assess whether vemurafenib-resistant cells exhibit chemotherapy resistance, we compared the sensitivity of parental and resistant primary and metastatic melanoma cells to cisplatin and etoposide (Figure 6).

Our results show that the primary WM793B-resistant melanoma cells displayed increased resistance to cisplatin in comparison to parental cells (IC_50_ (WM793B-P) = 1.586 ± 0.29 µM; IC_50_ (WM793B-R1) = 15.393 ± 5.31 µM; IC_50_ (WM793B-R2) = 12.066 ± 4.92 µM, *p* < 0.01 for *p* vs. R1 and *p* vs. R2) (Figure 6A). Apart from an increase in IC_50_, the efficacy of cisplatin was decreased during prolonged growth in vemurafenib-containing media. In contrast to total inhibition of viability in parental cells exposed to 100 μM cisplatin, the maximal inhibitory effect at 100 μM cisplatin was reduced from 92% in WM793B-R1 to 66% in WM793B-R2 cells (*p* < 0.001 for *p* vs. R2, *p* < 0.01 for R1 vs. R2).

The susceptibility of vemurafenib-resistant WM793B cells to etoposide was also reduced (Figure 6C). Although IC_50_ values for WM793B and WM793B-R1 were not significantly changed (IC_50_ (P) = 0.524 ± 0.26 µM; IC_50_ (R1) = 1.157 ± 0.77 µM), the maximal inhibition at 100 μM etoposide was reduced from 90% in parental cells to 36% in WM793B-R1 cells (*p* < 0.01). However, during prolonged growth in vemurafenib-containing media, the cytotoxic effect of etoposide was partially restored (maximal inhibition 19% at 100 μM etoposide, *p* < 0.01 for R1 vs. R2); however, the dose–response curve was shifted to the right (IC_50_ (R2) = 9.857 ± 4.43 µM, *p* < 0.01 for *p* vs. R2 and R1 vs. R2).

On the other hand, although a slight shift in IC_50_ was observed for A375M-R1 cells (IC_50_ (P) = 1.428 ± 0.76 µM; IC_50_ (R1) = 5.183 ± 2.43 µM, *p* < 0.05 for *p* vs. R1, one-way ANOVA and post hoc Tukey’s test; IC_50_ (R2) = 3.977 ± 1.78 µM), the resistant metastatic A375M cells showed similar sensitivity results to cisplatin as did the parental cells (Figure 6B). Moreover, the acquired resistance to vemurafenib did not change the sensitivity to etoposide in metastatic A375M melanoma cells (IC_50_ (P) = 0.424 ± 0.14 µM; IC_50_ (R1) = 0.570 ± 0.02 µM; IC_50_ (R2) = 0.593 ± 0.40 µM) (Figure 6D). The cross-resistance between vemurafenib and chemotherapy has been reported in the recent study by Erdmann et al., where they showed that the chronic exposure of melanoma cells to vemurafenib induced resistance to dacarbazine via PI3K/AKT/mTOR hyperactivation [51].

SCCs enter the non-proliferative (quiescent) G0/G1 cell cycle phase, exhibit low metabolic activity characterized by reduced proliferation rate and a condition of cell cycle arrest [52]. In order to investigate whether generated vemurafenib-resistant cells accumulate in G0/G1 phase, we performed cell-cycle analysis of vemurafenib-resistant and parental WM793B and A375M cell lines. Additionally, we treated the cells with etoposide, the antitumor agent that induces double-strand breaks and cell cycle arrest in the G2/M phase [53], to emphasize potential differences in cycling between parental and resistant cell lines.

**Figure 7:**
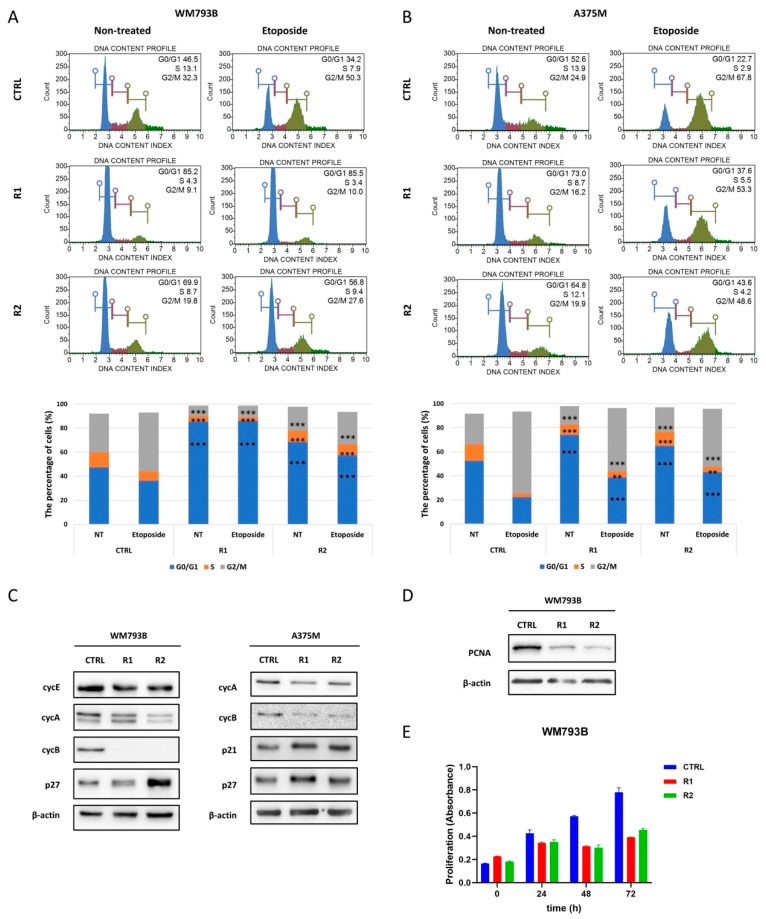
The cell-cycle analysis in the vemurafenib-resistant cell lines WM793B and A375M. The cell cycle phase distribution of parental (CTRL) and corresponding vemurafenib-resistant cells (R1 and R2) was analyzed by flow cytometry (**A**,**B**). The experiment was performed with untreated cells (NT) and after treatment with etoposide for 24 h. Statistical analysis was performed using MedCalc (v. 18.11.3). The normal distribution of continuous variables was confirmed using the D’Agostino–Pearson test, and the parametric statistical test one-way ANOVA with the Tukey–Kramer post hoc method was used. Asterisks indicate the statistical significance of each cell cycle phase in the resistant cell lines compared to the parental line. A *p*-value less than 0.01 is flagged with two stars (**), and a *p*-value less than 0.001 is flagged with three stars (***). Protein expression of cell cycle regulators (**C**) and PCNA proliferation marker (**D**) in parental (CTRL) and resistant (R1 and R2) cells. β-actin was used as a loading control. The proliferation rate of parental (CTRL) and resistant (R1 and R2) WM793B cells (**E**). The mean values of three biological replicates with standard deviations are shown.

The percentage of cells in G0/G1 was higher in non-treated vemurafenib-resistant cells compared to parental cells in both cell lines, connecting resistance with the slow-cycling state (Figure 7A,B). In addition, vemurafenib-resistant cells showed a lower percentage of G2/M cells after 24 h of exposure to etoposide compared to corresponding parental cells.

This effect was more prominent in WM793B-R cells, indicating a higher proportion of SCCs and thus higher resistance to etoposide treatment compared to parental WM793B cells (Figure 7A). Since parental WM793B cells showed slower accumulation in the G2/M phase after 24 h of etoposide treatment, WM793B-R cells were subjected to prolonged/extended etoposide treatment for 48 h (Supplementary Figure S1). Again, the percentage of G2/M cells after 48 h of etoposide treatment was lower in vemurafenib-resistant WM793B compared to parental cells.

The cell cycle progression is regulated by cyclins/cyclin-dependent kinases (CDKs). Therefore, to investigate the changes in the cell cycle progression in vemurafenib-resistant cells in more detail, we investigated the expression profile of cyclins. Cyclins are positive regulators of the cell cycle where cyclin E, in addition to cyclin D, regulates the phosphorylation of Rb, a tumor suppressor that contributes to the cell cycle checkpoint between the G1 and S phase [26]. Interestingly, we observed a reduced level of cyclin E in resistant WM793B compared to parental cells (Figure 7C).

In addition, the reduced levels of mitotic cyclins A and B, which drive progression through the S, G2, and M phases, were also observed in resistant WM793B and A375M cells compared to corresponding parental cells (Figure 7C). However, increased levels of cell cycle inhibitors p21 and p27 were detected in resistant A375M cells, while p27 was upregulated in resistant WM793B cells compared to corresponding parental cells (Figure 7C). The observation of the altered cell cycle profile in resistant cells prompted us to investigate the proliferation rate of resistant and parental primary WM793B melanoma cells. 

We noticed that the resistant WM793B cells exhibited a reduced level of a PCNA proliferation marker, and a lower proliferation rate compared to parental cells (Figure 7D,E), which is in line with the previous findings that have shown that the resistance to vemurafenib causes reduced cell growth in some melanoma cells lines [18]. To conclude, our results suggest that the resistant cells most likely transiently enter into cell cycle arrest at the G0/G1 phase and gain slow-cycling/non-proliferative quiescent/dormant cancer cell characteristics.

A recent study by M. Webster et al. shed new light on the role of the wild-type protein p53 in melanoma [54]. They showed that Wnt5A, a non-canonical Wnt ligand that drives a metastatic, treatment-resistant phenotype, stabilizes the half-life of p53 and uses p53 to initiate a slow-cycling state after stress (such as DNA damage, targeted therapy, or aging). In addition, a specific subset of drug-sensitive melanoma cells has been shown to enter a slow-cycling state that renders them resistant to targeted therapy. However, the inhibition of wild-type protein p53 blocks the slow-cycling phenotype and sensitizes previously resistant melanoma cells to BRAF/MEK inhibitors [54].

In our previous study, we analyzed the expression profiles of p53 and p73 protein isoforms in resistant melanoma cell lines and demonstrated that altered expression of the shorter isoforms of the p53 family can influence the aggressiveness of melanoma [55]. In another study of p53 expression in a subset of melanoma patient samples, we found a significant decrease in expression of Δ133p53β, an isoform typically associated with tumor invasion, increased cancer stem cell potential, and a poor prognosis [56,57,58]. Since increased expression of Δ133p53β is associated with breast tumor cell invasion [57], there is a possible association between decreased expression of this isoform in melanoma and reduced migration of our resistant melanoma cells.

### 2.6. Reduced Expression of NME Proteins in Vemurafenib-Resistant Melanoma Cell Line

NME1 and NME2 encode for A and B subunits of the nucleoside diphosphate kinases, which combine to form a series of homo- or heterohexameric isoenzymes (A6, A5B1, B6) [59]. Great interest for these enzymes was raised when it was discovered that NME1 was responsible for metastasis suppression in a murine melanoma model system [60]. This was followed by a series of studies linking decreased NME1 expression to aggressive properties of various tumor types [61]. The role of NME1 in melanoma has been thoroughly investigated and its potent effect as a suppressor of metastasis has been demonstrated in both cell lines and clinical melanoma tumor samples [62].

However, recent study showed that, in rare cases, NME1 can play a completely different role in melanoma. It was found to be responsible for the growth of melanomas that have aggressive tumor stem cell phenotype. Elevated levels of NME1 protein were found in a subpopulation of fast-cycling melanoma cells [63]. In addition, the same research group also indicated the existence of a rare subpopulation of cells with significantly reduced expression of NME1 in human melanoma cell lines that exhibit exceptional in vivo metastatic potential, confirming the role of NME1 protein as a metastasis suppressor [64]. The canonical function of NME1 as a metastasis suppressor has not been questioned; however, recent findings suggest that heterogenous expression of NME1 within melanoma can affect its activity.

**Figure 8:**
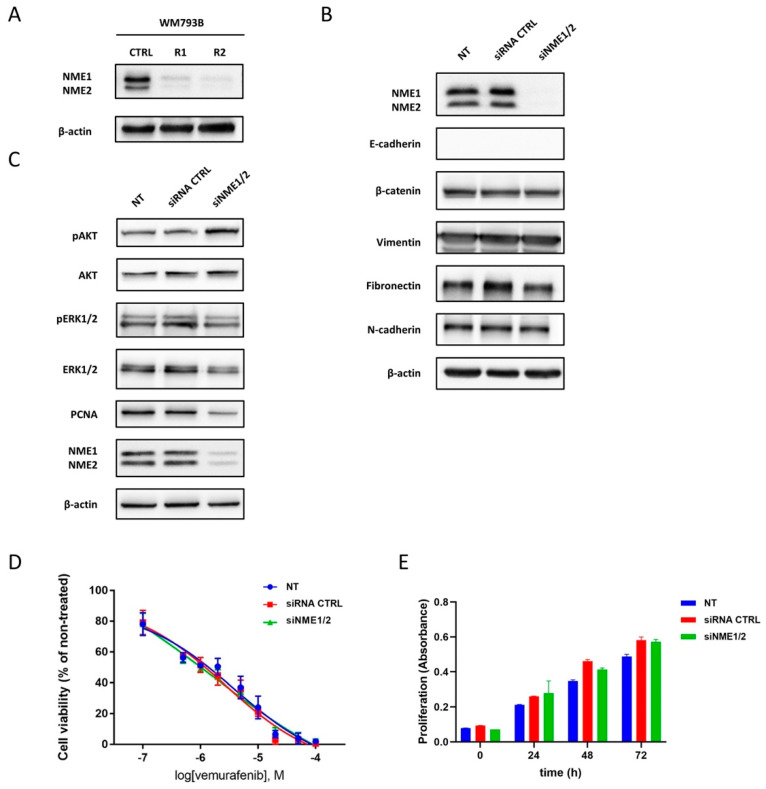
Silencing of NME1 and NME2 activates PI3K/AKT signaling pathway in WM793B cell line. Protein expression of NME1 and NME2 is reduced in vemurafenib-resistant WM793B cell line compared to parental line (**A**). Expression of EMT markers (**B**), signaling pathway, and proliferation markers (**C**) after silencing of NME1 and NME2 protein expression in WM793B cell line. Untreated samples (NT), samples transfected with the control siRNA (CTRL siRNA), and samples silenced with siNME1 and siNME2 (siNME1/2) were analyzed by western blot. β-actin was used as a loading control. A representative western blot from at least three experiments (three biological replicates) is shown. The viability of WM793B cells at increasing concentrations of vemurafenib was measured by MTT assay (**D**). The viability of untreated cells (NT), cells after transfection with control siRNA (CTRL siRNA), and cells after silencing of NME1 and NME2 protein expression (siNME1/2) is shown. The mean values of three biological replicates with standard deviations are shown. Each biological replicate was prepared in triplicate. The proliferation rate of untreated primary melanoma WM793B cells (NT), after transfection with control siRNA (CTRL siRNA), and cells after silencing of NME1 and NME2 protein expression (siNME1/2) (**E**). The mean values of two biological replicates with standard deviations are shown. Each biological replicate was prepared in triplicate.

Our study showed a drastic decrease in protein expression of NME1 and NME2 in vemurafenib-resistant primary melanoma cells (Figure 8A). The obtained results of reduced NME protein expression made us investigate the relationship with vemurafenib resistance in more detail. For that reason, we silenced the NME1 and NME2 protein expression in WM793B parental cells and examined the effects on the occurrence of vemurafenib resistance. Although silencing of both proteins over a 6-day period (required for MTT assay readout) was highly successful (Supplementary Figure S2), no effect on the resistance/sensitivity to vemurafenib was observed.

Both parental cells and cells with suppressed expression of NME1 and NME2 proteins showed comparable IC_50_ values (Figure 8D). Furthermore, the silencing of *NME1* and *NME2* did not affect the expression levels of fibronectin, N-cadherin, E-cadherin, vimentin, and β-catenin (Figure 8B). However, it has been shown that the NME1 protein negatively regulates MAPK and PI3K/AKT signaling pathways either through phosphorylation and thereby inactivation of KSR, a scaffold protein that facilitates the assembly of the kinases involved in the MAPK pathway [65], or by interacting with p110α, a PI3K catalytic subunit that can lead to negative regulation of p110α kinase activity and, thereby, the impaired activation of PI3K/AKT pathway [66].

In line with the previous findings [66,67], increased activity of PI3K/AKT signaling upon *NME1* and *NME2* silencing was detected (Figure 8C). However, the activity of MAPK signaling remained unchanged after *NME1* and *NME2* silencing in primary melanoma cells. As MAPK and PI3K/AKT are the main pathways that influence cell proliferation, growth, and differentiation, and are regulated by NME1 protein, we decided to investigate whether the reduced proliferation observed in resistant primary melanoma cells is caused by lower NME1 and NME2 levels.

To this end, we analyzed proliferation rate and the expression level of PCNA proliferation marker upon *NME1* and *NME2* silencing in the primary melanoma cell line. Although *NME* silencing did not influence the proliferation rate (Figure 8E), it reduced the level of PCNA in primary melanoma cells (Figure 8C), which is in agreement with the previous findings. For example, Wang and coauthors showed that *NME1* silencing does not affect the proliferation of melanoma cell lines when grown in monolayer cultures; however, it moderately reduced the level of Ki67 proliferation marker in 451Lu melanoma cell line [63].

To conclude, our results imply that the reduced level of NME1 and NME2 is most likely the result of vemurafenib-gained resistance and is one of the causes of increased PI3K/AKT signaling influencing some of the reported features of resistant primary cells. However, future studies are needed to identify the molecular regulators of NME1 and NME2 expression and consequently the changes in resistant melanoma cell phenotype.

## 3. Materials and Methods

### 3.1. Cell Culture

Primary WM793B (ATCC**^®^** CRL_2806™) and metastatic A375M (ATCC**^®^** CRL_1619™) human melanoma cell lines (both BRAF V600E) were used for the generation of vemurafenib-resistant sublines. Cell lines were kindly provided by Daniele Bergamaschi, Blizard Institute, UK. Both naive and vemurafenib-resistant cell lines were maintained in RPMI 1640 medium (Lonza, Basel, Switzerland) supplemented with 10% FBS (Thermo Fisher Scientific, Waltham, MA, USA), 100 units/mL penicillin (Sigma Aldrich, St. Louis, MO, USA), 100 µg/mL streptomycin (Sigma Aldrich, St. Louis, MO, USA), 2 mM L-glutamine (Sigma Aldrich, St. Louis, MO, USA), and 1 mM sodium pyruvate (Life Technologies, Carlsbad, CA, USA) in a humidified atmosphere with 5% CO2 at 37 °C. All cell lines were tested to be mycoplasma-free.

### 3.2. Establishment of Vemurafenib-Resistant A375M and WM793B Melanoma Cell Lines

To generate vemurafenib-resistant cell lines, A375M and WM793B cells were seeded at low density (~20% confluence). To generate A375M cells with acquired resistance to vemurafenib, the cells were propagated with gradually increasing concentrations (0.5, 0.75, 0.8, 0.9, and 1 µM) of vemurafenib (PLX4032, Sandoz, Holzkirchen, Germany) for 8 weeks, and the resistant cells were thereafter maintained in 1 or 2 µM vemurafenib (A375M-R1 and A375M-R2, respectively).

WM793B cells were treated with 3 µM vemurafenib for one week, and during the next 7 weeks, they were exposed to 4 µM vemurafenib. After 8 weeks of selection, they were considered resistant (WM793B-R) and propagated further in culturing medium containing 4 µM vemurafenib. The WM793B-R1 and -R2 differ in the length of vemurafenib treatment where R1 refers to a treatment of 2 to 7 months and R2 to a treatment of 7 to 12 months.

### 3.3. MTT Assay

The proliferation rate of melanoma cells, their resistance to vemurafenib, and the cytotoxic effects of cisplatin and etoposide were determined using colorimetric 3-(4,5-dimethylthiazol-2-yl)-2,5-diphenyl tetrazolium bromide (MTT) assay. The assay is based on the ability of live cells to cleave MTT to an insoluble formazan product due to the activity of mitochondrial dehydrogenases. Briefly, the number of cells that were seeded into 96-well plates was empirically determined for each cell type. To assess cell proliferation, cells were incubated for 24, 48, 72, and 96 h.

To determine the cytotoxic effects of vemurafenib, cisplatin, or etoposide, 24 h after plating melanoma cells were incubated with serial dilutions of chemotherapeutics for 72 h. For both tests, at the end of the treatment period, 40 μL of MTT solution prepared in RPMI-1640 medium (final concentration 0.5 mg/mL) was added to each well and incubated for 3 h at 37 °C. The precipitated formazan was dissolved in 160 μL of DMSO while the absorbance was recorded on a microplate reader at 570 nm. The IC_50_ values were calculated using GraphPad software (v. 7.04) and nonlinear regression (log(inhibitor) vs. response–variable slope, three or four parameter equation).

### 3.4. Migration (Wound Healing Assay and Culture Insert Assay)

For wound healing assay, the cells were seeded 24 h before the experiment in a 24-well plate. A straight line was scratched with a pipette tip to detach the confluent cells from the bottom of the well and washed with PBS to remove detached cells. A new medium was added, and the resulting gap image was captured under an EVOS FL microscope (Invitrogen, Thermo Fisher Scientific, Waltham, MA, USA) at the time of causing the wound as well as 8 and 24 h later. For video microscopy, the resulting gap was filmed using Inverted Olympus IX83 microscope (Olympus Corporation, Tokyo, Japan). The images were taken every 5 min in a period of 24 h. The results were processed using the ImageJ program (Rasband, W.S., U. S. National Institutes of Health, Bethesda, MD, USA, https://imagej.nih.gov/ij/). and displayed as a percentage of the closed area.

For the culture insert assay (Boyden-chamber-based), chambers with a membrane pore diameter of 8 μm were used (Falcon, Corning, NY, USA). In the upper chamber, 20,000 cells were seeded in 250 μL of medium without FBS. A complete medium (750 μL with FBS) was added to the lower chamber. The cells migrated for one hour after which the chambers were washed in PBS and stained in 750 μL of a 4 μg/mL solution of calcein for one hour. The initial migration experiments were performed at different time points (from 0.5 to 2.5 h), after which the optimal migration time of 1 h was selected for future experiments. Stained cells were imaged under a microscope, and the number of migrating cells was determined using ImageJ.

### 3.5. RNA Isolation and cDNA Synthesis

RNA was isolated using PureLink^®^ RNA Mini Kit (Ambion, Thermo Fisher Scientific, Waltham, MA, USA) including DNase treatment (Qiagen, Hilden, Germany) according to the manufacturer’s protocol. All steps of isolation were performed at 4 °C to prevent RNA degradation. Reverse transcription was performed using High Capacity cDNA Reverse Transcription Kit (Applied Biosystems, Thermo Fisher Scientific, Waltham, MA, USA) according to the manufacturer’s instruction in GeneAmp PCR System 2700 (Applied Biosystems, Thermo Fisher Scientific, Waltham, MA, USA) 10 min at 25 °C, 120 min at 37 °C, 5 min at 85 °C and hold at 4 °C. For qPCR analysis, cDNA was diluted to a concentration of 25 ng/μL.

### 3.6. Quantitative PCR (qPCR)

A total of 25 ng of cDNA was used for the qPCR gene expression analysis of *SNAIL*, *SLUG*, *TWIST*, *MMP2*, *MMP9*, *VIM*, and *FN1*. The primers were as follows: *SNAIL*: F: 5′-GCTGCAGGACTCTAATCCAGA-3′ R: 5′-ATCTCCGGAGGTGGGATG-3′; *SLUG*: F: 5′-TGGTTGCTTCAAGGACACAT-3′ R: 5′-GTTGCAGTGAGGGCAAGAA-3′; *TWIST*: F: 5′-CCGGAGACCTAGATGTCATTGT-3′ R: 5′-CCCACGCCCTGTTTCTTTGA-3′; *TBP*: F: 5′-CACGAACCACGGCACTGATT-3′ R: 5′-TTTTCTTGCTGCCAGTCTGGAC-3′; *MMP2*: F: 5′-CCCCAAAACGGACAAAGAG-3′ R: 5′-CACGAGCAAAGGCATCATCC-3′ *MMP9*: F: 5′-CACTGTCCACCCCTCAGAGC-3′ R: 5′-GCCACTTGTCGGCGATAAGG-3′ *VIM*: F: 5′-TGTCCAAATCGATGTGGATGTTT-3′ R: 5′-TTGTACCATTCTTCTGCCTCCTG-3′ *FN1*: F: 5′-CCACCCCCATAAGGCATAGG-3′ R: 5′-GTAGGGGTCAAAGCACGAGTCAT-3′ *RPLP0*: F: 5′-GGCACCATTGAAATCCTGAGTGATGTG-3′ R: 5′-TTGCGGACACCCTCCAGGAAGC-3′.

The qPCR analysis was performed using Takyon Low Rox SYBR MasterMix dTTP Blue (for *SNAIL*, *SLUG*, *TWIST*, and *TBP*; Eurogentec, Seraing, Belgium) or SsoAdvanced Universal SYBR Green Supermix (for *MMP2*, *MMP9*, *VIM*, *FN1*, and *RPLP0*; BioRad Laboratories, Hercules, CA, USA) and quantified with the CFX96 Real-Time PCR Detection Systems (Bio-Rad Laboratories, Hercules, CA, USA) instrument under following cycling conditions for *SNAIL*, *SLUG*, *TWIST* and *TBP*: initial denaturation for 3 min at 95 °C, 40 cycles of 95 °C 15 s, 63 °C 20 s, and 72 °C 10 s, with the final step of 72 °C 10 s; and for *MMP2*, *MMP9*, *VIM*, *FN1*, and *RPLP0*: initial denaturation 95 °C 3 min, followed by 40 cycles of denaturation at 95 °C 10 s and annealing/elongation at 61 °C 30 s.

The results were analyzed with CFX Manager Software v3.1 (Bio-Rad Laboratories, Hercules, CA, USA), first normalized with Ct values of *TBP* for *SNAIL*, *SLUG*, and *TWIST* and Ct values of *RPLP0* for *MMP2*, *MMP9*, *VIM*, and *FN1*, and then normalized with Ct values of non-treated (parental) samples so that antilog values of 2^−∆∆Ct^ were presented as bars using GraphPad Prism v. 7.04 (GraphPad Software, San Diego, CA, USA).

### 3.7. Silencing of NME1 and NME2

Transient transfection of cells with small interfering RNA molecules (siRNA) was used for silencing the *NME1* and *NME2* genes. By direct silencing in adherent cells, we performed transfection with two siRNAs (ON-TARGETplus Human NME1 siRNA-Smartpool L-006821-00-0005 and ON-TARGETplus Human NME2 siRNA-Smartpool L-005102-00-0005, Dharmacon, Lafayette, CO, USA) using DharmaFECT 4 (Dharmacon, Lafayette, CO, USA) transfection reagent according to the manufacturer’s protocol. In parallel, we also transfected the cells with non-targeting, control siRNA (ON-TARGETplus non-targeting control siRNA#1, Dharmacon, Lafayette, CO, USA).

To determine the efficacy of silencing by western blot, cells were harvested 48 h after transfection. A portion of the cells was reseeded and collected 96 h later to verify the persistence of the silencing over a longer period required for the MTT assay.

### 3.8. Cell-Cycle Assay

For cell-cycle analysis by flow cytometry, 2 × 10^5^ cells per well were seeded in a 6-well plate one day before the etoposide treatment. After 24 h the cells were trypsinized and centrifuged for 5 min at 300× *g*. The supernatant was discarded, washed in PBS and the centrifugation step was repeated. The cell pellet was resuspended in 50 μL of PBS and added dropwise to previously prepared tubes with 1 mL of ethanol at 4 °C on a vortex device.

The tubes were stored at −20 °C. The next day, 200 μL of cell suspension was transferred to a clean tube, centrifuged for 5 min at 300× *g* and the supernatant was discarded. Cells were resuspended in 500 μL of PBS and repeated the centrifugation step after which the cells were resuspended in 200 μL of Muse Cell Cycle Reagent (Merck Millipore, Burlington, MA, USA) and incubated for 30 min at room temperature protected from light. Cells were analyzed on a Muse Cell Analyzer (Merck Millipore, Burlington, MA, USA).

### 3.9. Fluorescent Staining and Confocal Imaging

For fluorescent staining with phalloidin, the cells were seeded on eight-well glass bottom slides (Ibidi, Gräfelfing, Germany) with a removable silicone chamber, and after 24 h washed with PBS, fixed in 2% formaldehyde for 10 min at room temperature and washed with PBS again. After fixation, cells were permeabilized with Triton X-100 solution followed by PBS rinsing. For staining the cytoskeleton cells were incubated in 50 μg/mL solution of phalloidin TRITC (Sigma, Munich, Germany, P-1951) in PBS for 40 min at room temperature. After washing with PBS, cells were mounted in a mounting medium (DAKO, Glostrup, Denmark) supplemented with 1 µg/mL DAPI (Sigma, Munich, Germany) for nuclear staining.

Confocal microscopy was performed using Leica TCS SP8 X FLIM microscope equipped with an HC PL APO CS2 63×/1.40 oil objective, 405-nm diode laser, and a supercontinuum excitation laser (Leica Microsystems, Wetzlar, Germany). The excitation wavelengths and detection ranges used for confocal imaging were 405 nm and 430–500 nm for DAPI and 545 nm and 555–605 nm for TRITC. The hybrid (HyD) detectors were operated in the gated mode in order to suppress parasite reflection from the bottom glass surface of the cell-culture dish.

### 3.10. Protein Isolation and Western Blot

Proteins were extracted in Dulbecco’s phosphate buffer saline (DPBS) (Sigma Aldrich, St. Louis, MO, USA) completed with protease inhibitors (cOmplete, Mini, EDTA-free Protease Inhibitor Cocktail; Roche Diagnostics, Basel, Switzerland). Pellets were sonicated (1 mm probe, 2 × 10 s), and the protein concentration was determined using a Pierce BCA Protein Assay Kit (Pierce, Thermo Fisher Scientific, Waltham, MA, USA). After isolation, 30 μg of total proteins were separated on 8%, 10%, or 12% SDS-polyacrylamide gel and transferred to a nitrocellulose membrane (Merck Millipore, Burlington, MA, USA).

The antibodies used in this study were as follows: primary mouse anti-AKT (Cell Signaling, Danver, MA, USA, #2920, 1:1000), primary rabbit anti-pAKT (Cell Signaling, Danver, MA, USA, #9271, 1:200), primary rabbit anti-ERK1 (Santa Cruz, Dallas, TX, USA, sc-94, 1:1000), primary mouse anti-pERK (Santa Cruz, Dallas, TX, USA, sc-7383, 1:1000), primary mouse anti-ß-catenin (Sigma Aldrich, St. Louis, MO, USA, C7207, 1:1000), primary mouse anti-N-cadherin (BD Biosciences, Franklin Lakes, NJ, USA, 610920, 1:500), primary mouse anti-vimentin (Santa Cruz Biotechnology, Dallas, TX, USA, sc-32322, 1:1000), primary mouse anti-fibronectin (Santa Cruz Biotechnology, Dallas, TX, USA, sc-8422, 1:200), primary mouse anti-ß-actin (Proteintech, Rosemont, IL, USA, 60008-1-1g, 1:3000), primary rabbit anti-NME1/NME2 (kindly provided by late Dr. Ioan Lascu, University of Bordeaux, France; and Dr. Siniša Volarević, University of Rijeka, Croatia; 1:3000), primary rabbit anti-cyclin A (Santa Cruz, Dallas, TX, USA, sc-751, 1:500), primary mouse anti-cyclin B (Santa Cruz, Dallas, TX, USA, sc-245, 1:500), primary mouse anti-cyclin E (Santa Cruz, Dallas, TX, USA, sc-247, 1:400), primary rabbit anti-PCNA (Cell Signaling, Danver, MA, USA #13110, 1:1000), primary rabbit anti-p21 (Santa Cruz, Dallas, TX, USA, sc-397, 1:300), primary mouse anti-p27 (Santa Cruz, Dallas, TX, USA, sc-53871, 1:300), secondary horseradish peroxidase (HRP)-conjugated anti-rabbit (Cell signaling, Danver, MA, USA, #7074, 1:5000), and secondary HRP-conjugated anti-mouse (Cell signaling, Danver, MA, USA, #7076, 1:5000). Proteins were visualized using Western Lightning Chemiluminescence Reagent Plus (Perkin Elmer, Waltham, MA, USA) on Alliance 4.7 imaging system (UVItec, Cambridge, UK).

### 3.11. Statistical Analysis

Statistical analysis was performed using GraphPad Prism (v. 7.04) and MedCalc (v. 18.11.3) programs. All experiments were done in triplicates. The statistical test used in the analysis is shown in the description of each figure, and statistically significant results are marked by an asterisk (*). A *p*-value less than 0.05 is flagged with one star (*), a *p*-value less than 0.01 is flagged with two stars (**), a *p*-value less than 0.001 is flagged with three stars (***), and if a *p*-value is less than 0.0001, it is flagged with four stars (****).

## 4. Conclusions

Our study revealed that in vitro-generated vemurafenib-resistant melanoma cells exhibit specific features of slow-cycling cells, which show some morphological and molecular features of EMT-like cells, increased resistance to chemotherapy, altered levels of cell-cycle regulators, and consequently reduced proliferation. Other characteristics of vemurafenib-resistant cells included increased collective migration and reactivation of MAPK or activation of PI3K/AKT signaling pathways depending on the cell line.

In addition, the PI3K/AKT signaling pathway was shown to be regulated by NME1 and NME2 metastasis suppressors in the WM793B primary melanoma cell line. To the best of our knowledge, this is the first study that reported a reduced level of NME1 and NME2 proteins as a result of vemurafenib treatment. Future studies are needed to elucidate the mechanism of vemurafenib-dependent suppression of NME metastasis suppressor proteins and the association with PI3K/AKT signaling.

## Data Availability

Data are contained within the article and Supplementary Materials.

## Supplementary Materials

The supporting information can be downloaded at: https://www.mdpi.com/article/10.3390/ijms23179910/s1.
