# Supplementary material for: Characterization of Vemurafenib-Resistant Melanoma Cell Lines Reveals Novel Hallmarks of Targeted Therapy Resistance
Source: Int J Mol Sci. 2022 Aug 31;23(17):9910. doi: 10.3390/ijms23179910 (PMC9455970; doi:10.3390/ijms23179910)
Supplement: Supplementary file 1 [file ijms-23-09910-s001.zip › ijms-1850608-supplementary Proofed.pdf]

Supplementary material

Characterization of vemurafenib-resistant melanoma cell lines reveals novel hallmarks of targeted therapy resistance

Submitted to **International Journal of Molecular Sciences, Special Issue "Molecular Biology of Melanoma 2.0"**

Authors: Martina Radić, Ignacija Vlašić, Maja Jazvinšćak Jembrek, Anđela Horvat, Ana Tadijan, Maja Sabol, Marko Dužević, Maja Herak Bosnar and Neda Slade

Corresponding authors:

Neda Slade; E-mail: slade@irb.hr

Maja Herak Bosnar; E-mail: mherak@irb.hr

Supplementary Figures and Figure Legends:

Figure S1. **The cell cycle in the vemurafenib-resistant cell lines WM793B.** The distribution of the parental (CTRL) and the corresponding vemurafenib-resistant cells (R1 and R2) according to cell cycle phases was analyzed by flow cytometry. The experiment was performed with untreated cells (NT) and after the treatment with etoposide for 48. Statistical analysis was performed in MedCalc (v. 18.11.3). The normal distribution of continuous variables was confirmed using the D'Agostino–Pearson test, and the parametric statistical test one-way ANOVA with the Tukey Kramer post-hoc method was used. Asterisks indicate the statistical significance of each cell cycle phase in the resistant cell lines compared to the parental line. Statistically significant results are marked by an asterisk (*). A p-value less than 0.05 is flagged with one star (*), a p-value less than 0.01 is flagged with two stars (**), and p-value less than 0.001 is flagged with three stars (***).

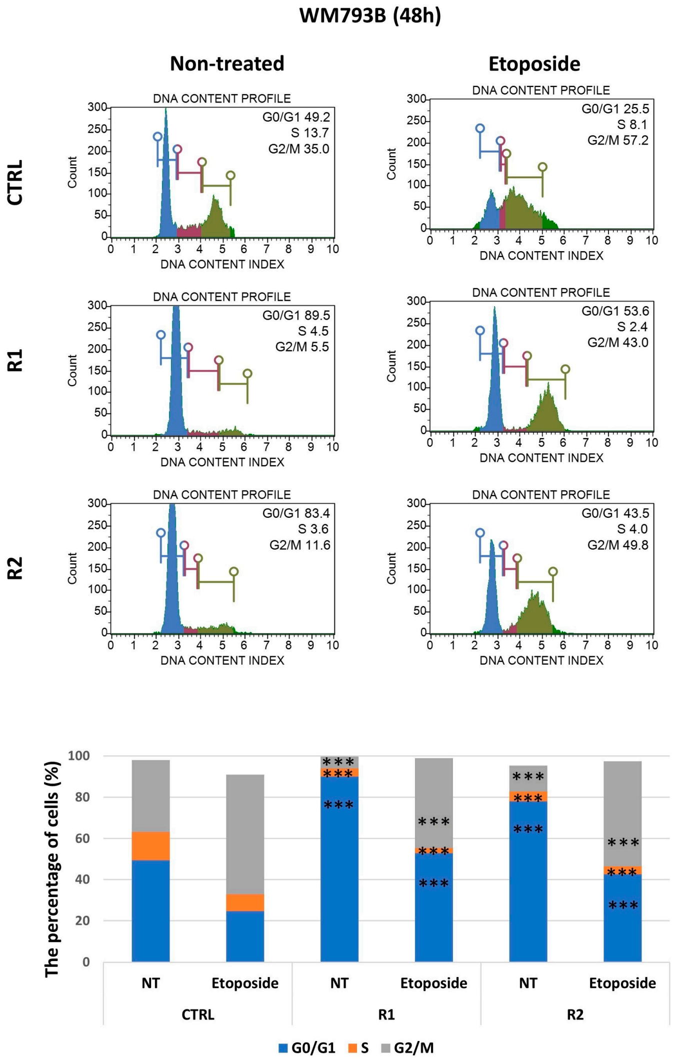

Figure S2. Silencing efficacy of siRNAs targeting NME1 and NME2. Silencing efficacy was determined at the time cells were seeded for MTT assay (48 hours after silencing) and at the time MTT assay was measured (48 + 96 hours after silencing). Untreated samples (NT) and samples silenced with control siRNA (siRNA CTRL) are shown. β-actin was used as a loading control.

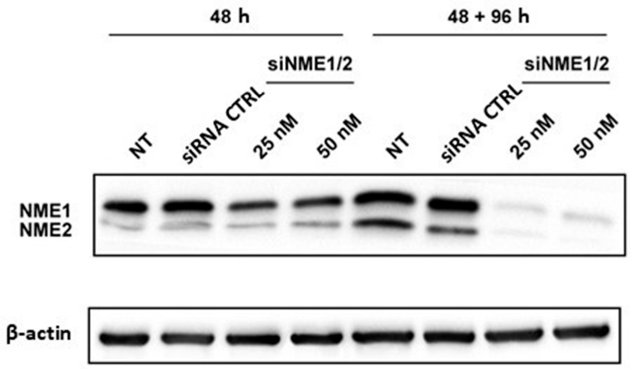

Supplementary Video:

Video S1: Phase-contrast time-lapse microscopy of wound-healing migratory assay. Phase-contrast time-lapse microscopy of wound-healing migratory assay of parental A375M cells (**A**), of resistant A375M R1 cells (**B**), and of resistant A375M R2 cells (**C**).
